# A novel study on bean common mosaic virus accumulation shows disease resistance at the initial stage of infection in *Phaseolus vulgaris*

**DOI:** 10.3389/fgene.2023.1136794

**Published:** 2023-03-20

**Authors:** Ali Çelik, Orkun Emiralioğlu, Mehmet Zahit Yeken, Vahdettin Çiftçi, Göksel Özer, Yoonha Kim, Faheem Shehzad Baloch, Yong Suk Chung

**Affiliations:** ^1^ Department of Plant Protection, Faculty of Agriculture, Bolu Abant Izzet Baysal University, Bolu, Türkiye; ^2^ Department of Field Crops, Faculty of Agriculture, Bolu Abant Izzet Baysal University, Bolu, Türkiye; ^3^ Laboratory of Crop Production, Department of Applied Biosciences, Kyungpook National University, Daegu, Republic of Korea; ^4^ Faculty of Agricultural Sciences and Technologies, Sivas University of Science and Technology, Sivas, Türkiye; ^5^ Department of Plant Resources and Environment, Jeju National University, Jeju, Republic of Korea

**Keywords:** BCMV, real-time PCR, SYBR green, common bean, viral load

## Abstract

Accurate and early diagnosis of bean common mosaic virus (BCMV) in *Phaseolus vulgaris* tissues is critical since the pathogen can spread easily and have long-term detrimental effects on bean production. The use of resistant varieties is a key factor in the management activities of BCMV. The study reported here describes the development and application of a novel SYBR Green-based quantitative real-time PCR (qRT-PCR) assay targeting the coat protein gene to determine the host sensitivity to the specific NL-4 strain of BCMV. The technique showed high specificity, validated by melting curve analysis, without cross-reaction. Further, the symptoms development of twenty advanced common bean genotypes after mechanical BCMV-NL-4 infection was evaluated and compared. The results showed that common bean genotypes exhibit varying levels of host susceptibility to this BCMV strain. The YLV-14 and BRS-22 genotypes were determined as the most resistant and susceptible genotypes, respectively, in terms of aggressiveness of symptoms. The accumulation of BCMV was analyzed in the resistant and susceptible genotypes 3, 6, and 9 days following the inoculation by the newly developed qRT-PCR. The mean cycle threshold (Ct) values showed that the viral titer was significantly lower in YLV-14, which was evident in both root and leaf 3 days after the inoculation. The qRT-PCR thus facilitated an accurate, specific, and feasible assessment of BCMV accumulation in bean tissues even in low virus titers, allowing novel clues in selecting resistant genotypes in the early stages of infection, which is critical for disease management. To the best of our knowledge, this is the first study of a successfully performed qRT-PCR to estimate BCMV quantification.

## 1 Introduction

Plant viruses are obligate intracellular pathogens that inhabit the symplast of their hosts solely to accomplish their replication process, including transcription/translation, encapsidation, and transportation to the new host’s cells. The viral accumulation in the various parts of a host plant is crucial for developing symptoms and persistence of the virus in the plant (Hipper et al., 2013). The interaction between viruses and their host plants has intrigued researchers working on breeding programs to improve resistant varieties. Developing new resistant varieties against viral pathogens in plant breeding programs is one of the most important approaches to coping with new environmental challenges lived in agriculture and making the world more liveable and self-sufficient (Borrelli et al., 2018). This phenomenon is that increasing disease resistance in plants plays a significant role in regulating crop production to meet the demand of the increasing global population.

Legumes, known as an important food source in human nutrition on a global scale, have been infected by more than 168 plant viruses from 16 families (Chatzivassiliou, 2021). Bean common mosaic virus (BCMV), a member of *Potyvirus*, is one of the most important viral pathogens of common bean and has an economic impact on crop production worldwide. The pathogen is monopartite flexuous rod-shaped with a positive sense ssRNA genome of about 10 kb size, transmitted by seeds and aphids (El-Sawy et al., 2014). The infection rate of BCMV by seed transmission may vary between 15 and 30% depending on host genotype, viral strain, infection time, and cultivating conditions (Morales, 1989; Arlı-Sökmen et al., 2016). Virus-free seeds are recommended for the management of BCMV; however, the use of resistant varieties is considered to be the most effective and practical control method (Drijfhout, 1978; Hall, 1994; Miklas et al., 2000). Bean resistance to BCMV is managed by the dominant *I* gene and six different recessive genes (*bc-u, bc-1/bc-1* ^*2*^ *, bc-2/bc-2* ^*2*^ *,* and *bc-3*) (Drijfhout, 1978; Kelly et al., 1995).

Research on recent developments in the understanding of host fitness for resistance is still ongoing (Soler-Garzón et al., 2021). Based on the pathogenicity (P0, P1, P1^2^, P2, P2^2^) and differential bean host reaction, BCMV isolates were classified into 7 pathogroups (PGs), including PGI-II-IV-V-VI-VII and VIII (Drijfhout, 1978; Feng et al., 2014; Feng et al., 2015; Kyrychenko et al., 2019). Due to the necessity of using resistant varieties, plant breeders need to investigate sources of BCMV resistance in response to the challenges of common bean susceptibility. Several studies have been conducted to evaluate the existence of disease-resistant genotypes (Mukeshimana et al., 2005; Bello et al., 2014; Boersma et al., 2014; Yeken et al., 2018; Palacıoğlu et al., 2021).

The detection of BCMV in different plant tissues was carried out with several serological and molecular methods, including Double Antibody Sandwich Enzyme-Linked Immunosorbent Assay (DAS-ELISA) (Vetten et al., 1992), reverse transcription-polymerase chain reaction (RT-PCR) (Xu and Hampton, 1996), and sequencing analysis (Arlı-Sökmen et al., 2016). Effective and highly sensitive approaches have been improved in recent years due to the giant breakthroughs in biotechnology and molecular virology (Yadav et al., 2016). One of these advances is quantitative real-time PCR (qRT-PCR), which is increasingly being used to develop sensitive, accurate, and reliable detection in plant virology (Poojari et al., 2016; Malandraki et al., 2017). The qRT-PCR is based on displaying and calculating the increasing fluorescent radiation (cycle threshold, Ct) based on the amplification of the target region at each cycle (Boonham et al., 2014), showing that low Ct levels indicate higher virus titre and high Ct less viral load, respectively. The qRT-PCR assay has many advantages due to its simplicity, requiring short hands-on time, and automated amplified capacity of detected samples (Espy et al., 2006). The diagnosis of various plant viruses has been accomplished by several studies based on qRT-PCR assays (Mumford et al., 2000; Picó et al., 2005; López et al., 2006; Ling et al., 2007; Hongyun et al., 2008; Ruiz-Ruiz et al., 2009a; Debreczeni et al., 2011; Ferriol et al., 2011; Sharma and Dasgupta, 2012; Herrera-Vásquez et al., 2015; MacKenzie et al., 2015). In addition to diagnostic studies, some significant progress have also been performed on qRT-PCR assays to detect mixed plant viruses infections (Mortimer-Jones et al., 2009; Abrahamian et al., 2013), in cross-protection studies (Ruiz-Ruiz et al., 2009b; Hanssen et al., 2010), in determining virus-vector association and epidemic analysis (Olmos et al., 2005; Rotenberg et al., 2009; Ferriol et al., 2013; Debreczeni et al., 2014), in the correlation between virus accumulations and plant resistance (Gil-Salas et al., 2009; Galipienso et al., 2013; Soler et al., 2015), and in the discrimination mutations caused by aggressive strains (Carrasco et al., 2007; Peña et al., 2014).

Virus quantification is the process of quantifying the amount of viral load in a given volume to calculate the virus concentration. Quantification of a virus enables to detection of its absolute accumulation rather than the presence/absence of the pathogen, so that protective measures can be taken at an early stage (Rubio et al., 2020). Quantification studies conducted by real-time PCR are generally carried out using a hydrolysis probe (TaqMan) or SYBR Green fluorescence dye (Kokane et al., 2021). Comparatively, SYBR Green-based real-time PCR is a cheaper reporter system than a probe due to binding to the small channel of double-stranded DNA and emits fluorescence associated with the quantification of amplified products existing in the target (Levin, 2004; Kokane et al., 2021). Several methods have been developed to quantify many organisms infecting different hosts using qRT-PCR (Santhosh et al., 2007; Bhullar et al., 2014; Mahmoudian et al., 2011; Bayraktar et al., 2016; Özer and Bayraktar, 2019; Olveira et al., 2021). Recently, identification and quantification studies by qRT-PCR were carried out by Chung et al. (2021) on patients infected with SARS-COV-2 (COVID-19). Several plant viruses have also been quantified using the qRT-PCR approach, including cucumber vein yellowing virus (CVYV) (Picó et al., 2005), barley yellow dwarf virus (BYDV) (Jarosová and Kundu, 2010), wheat yellow mosaic virus (WYMV) (Liu et al., 2013), different grapevine viruses (Fajardo et al., 2017), tomato spotted wilt virus (TSWV) (Soler et al., 2015), and cassava brown streak disease (CBSD) (Shirima et al., 2017). However, no study is available in the literature for the detection and quantification of BCMV in host tissues based on qRT-PCR assay.

The present study aimed to; i) identify resistance sources of BCMV NL-4 (PGVII) strain in the genetic germplasm using pathogenicity tests, ii) develop an SYBR Green-based qRT-PCR assay targeting the coat protein (CP) sequence of a BCMV NL-4 strain to assess the accumulation of the virus in various plant tissues, and iii) evaluate this new technique for selection resistant genotypes at early stages of viral infection. The result of the study would help breeders who are improving bean resistance and virologists trying to understand the host-pathogen interactions.

## 2 Materials and Methods

### 2.1 Virus source and plant material

The isolate of BCMV described as NL-4 strain was maintained in the common bean cultivar Kantar-05. The BCMV infection on the host plant was confirmed by conventional RT-PCR and partially sequenced through the coat protein gene as described in Traykova et al. (2008). Twenty common bean genotypes (Supplementary Table S1), which were previously characterized in terms of morphologically and agronomically according to IPGRI (International Plant Genetic Resources Institute) and EU-CPVO (European Union Community Plant Variety Office), were used in the study for pathogenicity tests. The cultivars Kantar-05 and Yakutiye-98, provided by the Research Institutes of the Republic of Turkey Ministry of Agriculture and Forestry, were included in the experiment as susceptible and resistant check lines for BCMV NL-4 strain, respectively (Sökmen et al., 2012).

### 2.2 Virus inoculation, experimental design, and evaluation of disease severity

All genotypes were planted in 20 cm pots containing a sterilized mixture of soil, perlite, and sand (1:1:1, v:v) and maintained in a growth chamber under a 16:8 h L:D photoperiod at 25°C and 70% humidity. The mechanic inoculation was carried out with 3 replications, following full deployment of the cotyledon leaves of 10-day-old plants, of which the first trifoliate leaves were observed. A total of 0.3 g of an infected leaf sample was ground in a mortar with a pestle with 2 ml of PBS (4°C) and then used to mechanical inoculate leaves dusted with carborundum powder. Inoculated plants were inspected daily for symptoms during the experiment. After 25 days post-inoculation (pi), symptoms on common bean genotypes were assessed using a 0–3 scale: 0 = no symptoms, 1 = mild foliar symptoms, 2 = moderate foliar symptoms, and 3 = severe distortion and malformation, which was developed by Odu et al. (2004) for *Potyviruses*. Two genotypes were selected as the most susceptible and resistant after the pathogenicity assay for further studies.

### 2.3 Preparing hoagland solution and sampling plant material for qRT-PCR analyses

**FIGURE 1:**
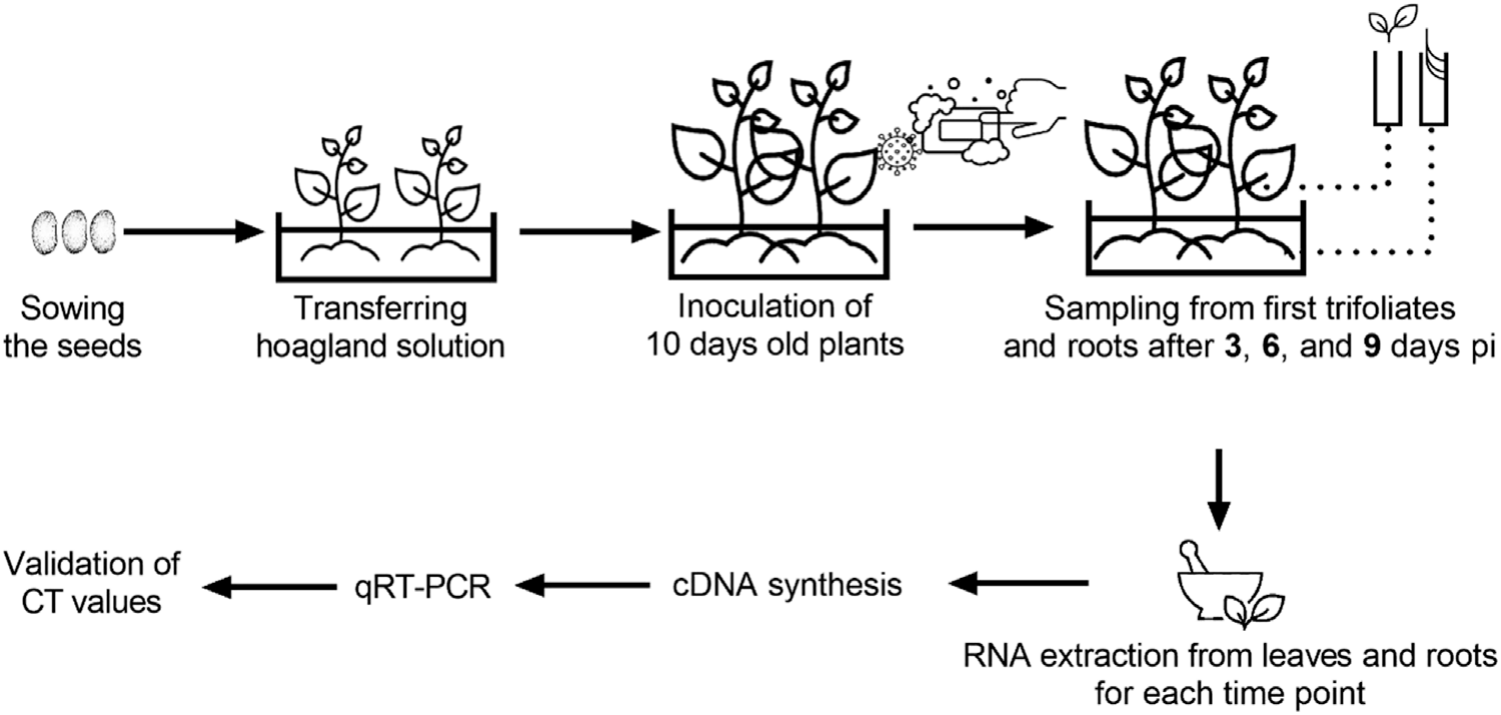
Detailed schematic representation of experimental design and sampling.

Seeds of two genotypes selected by the pathogenicity assay were surface sterilized in a 0.5% NaOCl solution for 2 min and placed on two layers of filter paper in 100 × 15 mm plastic Petri dishes containing 5 ml of sterile distilled water. After 4 days, pre-germinated seeds were transferred to a pot filled with Hoagland nutrient solution (Epstein, 1972). Both genotypes were inoculated with the virus isolate and kept in a growth room under the conditions described above. The nutrient solution was refreshed in 48 h periodically. Samples of fully developed first trifoliate leaves and roots were collected from inoculated and control plants at 3-, 6-, and 9-days pi and immediately transferred into liquid nitrogen. The samples were kept at −132°C for long-term storage after RNA isolation. A detailed schematic representation of experimental design and sampling is given in Figure 1.

### 2.4 Primer design for qRT-PCR

The coat protein sequences of the BCMV NL-4 isolate used in all biological experiments were obtained according to Traykova et al. (2008) and deposited in GenBank under an accession number (OL741709). When designing primers, the 362 bp length coat protein sequence of BCMV NL-4 was used as input data for the NCBI primer design tool (https://www.ncbi.nlm.nih.gov/tools/primer-blast/). The tool-generated primers (Supplementary Table S2) were manually selected based on amplicon size, melting temperature, position, GC content, and annealing temperatures. The results were checked on the Beacon Designer (http://free.premierbiosoft.com) and mFold (http://www.idtdna.com/Scitools/Applications/mFold/) platforms. Comparative controls were also attempted using the BLASTn on NCBI primer design tool to check the specificity of the primers with other related common bean infecting *Potyvirus*, bean common mosaic necrosis virus (BCMNV), and probable hosts of BCMV to reduce the cross-reactivity due to sequence similarities.

### 2.5 RNA extraction, DNase treatment, and cDNA synthesis

Total RNA was extracted from 150 mg of infected leaves and roots using NucleoZOL RNA extraction solution (MACHEREY-NAGEL GmbH & Co., Dueren, Germany) according to the manufacturer’s instructions. A total of 8 µg RNA was treated with DNase I (RNase-free, supplied with MnCl_2_) (Thermo Fischer Scientific, Waltham, MA, United States) according to the manufacturer’s instructions. Complementary DNA (cDNA) was synthesized from 2 µg of total RNA using Thermo Scientific RevertAid First Strand cDNA Synthesis Kit (Thermo Fischer Scientific, Waltham, MA, United States) in a final volume of 20 µL based on the manufacturer’s instructions with the addition of oligo (dT)18 primer that anneals selectively to the poly (A) tail of viral RNA. Diluted cDNA samples were stored at −20°C for further quantification studies. Nucleic acid measurements were conducted with a DS-11 FX + series spectrophotometer (Denovix Inc., Wilmington, DE, United States).

### 2.6 SYBR green qRT-PCR and primer efficiency

The qRT-PCR was performed in the CFX Connect Real-Time PCR System (Bio-Rad, Hercules, CA, United States) using a 25 μL reaction mix, including 12.5 μL of RealQ Plus 2x Master Mix Green without ROX (Ampliqon, Odense, Denmark), 0.75 μL each of primers (10 μM), 1 μL of cDNA, and 10 μL of RNase-free ddH2O. The reaction was carried out under the following conditions: one cycle at 95°C for 15 min for activation of the TEMPase hot-start enzyme, followed by 40 cycles of 95°C for 15 s, 61°C for 45 s, and 72°C for 20 s. The Ct values were calculated by the CFX Maestro Software (Bio-Rad, Hercules, CA, United States). The samples were subjected to melting curve analysis by heating the samples from 65 to 95°C following the final cycle of the PCR to detect specific and non-specific PCR products. Each sample was examined using three technical duplicates, and all plates contained virus-infected positive (with Kantar-05 and Yakutiye), healthy plant RNA, and no template samples as controls. The five-fold cDNA dilution series was used to determine the primer efficiency and standard curves. The qRT-PCR experiment was performed according to the conditions described above.

### 2.7 Data analyses

The analysis of variance (ANOVA) was analysed for disease severity for all genotypes and CT values of resistant and sensitive genotypes at different time points and followed by Least Significant Test at *p* ≤ 0.01 with SAS Statistical Software (SAS Institute Inc., Cary, NC, United States). Then, the data was transferred to GraphPad Prism version 6.04 for Windows (GraphPad Software, San Diego, CA, United States) and visualized as graphics.

## 3 Results

### 3.1 Host suitability

**FIGURE 2:**
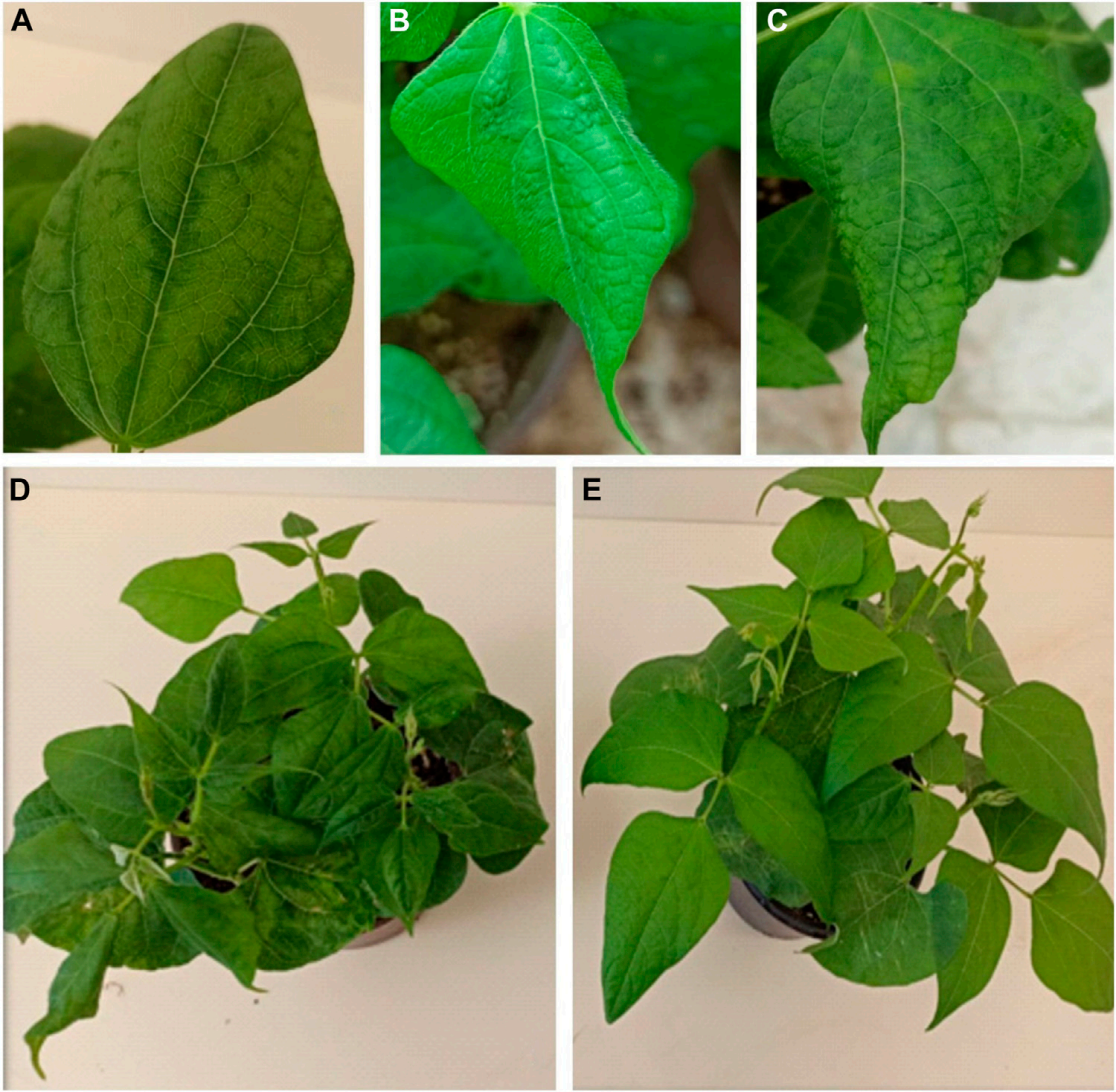
Different types of symptoms observed on common bean leaves 25 days pi BCMV NL-4. **(A)**: mosaic symptom, **(B,C)**: wrinkling symptoms with mosaic, **(D)**: dwarfing with severe distortion and malformation on BRS-22, **(E)**: no visible symptoms on YLV-14.

Various symptoms, including dwarfing, wrinkling, and mosaic, were observed on common bean genotypes, confirming BCMV infection (Figures 2A–C). The BRS-22 genotype showed the first symptoms caused by the viral inoculation at 14 days pi, and severe mosaic symptoms on this genotype were observed at 25 days pi (Figure 2D). However, no viral symptoms were observed in the genotype YLV-14 due to BCMV infection (Figure 2E). Mild foliar symptoms occurred on 7 genotypes, while moderate foliar and wrinkling symptoms were observed on 11 genotypes. All genotypes were symptomatic (except for YLV-14), and the results of statistical analyses also showed that the disease severity level of YLV-14 and BRS-22 were similar to those recorded for resistance (Yakutiye-98) and susceptible (Kantar-05) check lines, respectively. The classification of common bean genotypes and incidence of symptom types were presented in Table 1. Therefore, BRS-22 and YLV-14 were found to be the most susceptible and resistant genotypes against BCMV NL-4 based on pathogenicity tests and selected for quantification studies.

**TABLE 1:** Classification of common bean genotypes based on symptom severity.

Genotype	Symptoms	Disease severity*	Group
BLCK-7	Moderate foliar, wrinkling	1.67^bc^	2
BLKSR-3	Mild foliar	1.00^cd^	1
BLKSR-4	Moderate foliar, wrinkling	1.67^bc^	2
BLKSR-19	Moderate foliar, wrinkling	2.33^ab^	2
BRS-3	Moderate foliar, wrinkling	2.33^ab^	2
BRS-4	Moderate foliar, wrinkling	1.67^bc^	2
BRS-21	Mild foliar	1.00^cd^	1
BRS-23	Mild foliar	1.33^bcd^	1
BRS-22	Severe disortion and malformation	3.00^a^	3
BRS-24	Mild foliar	1.00^cd^	1
DZC-2	Moderate foliar, wrinkling	2.33^ab^	2
DZC-3	Moderate foliar, wrinkling	2.33^ab^	2
ÇNK-2	Moderate foliar, wrinkling	2.00^abc^	2
ÇNK-4	Moderate foliar, wrinkling	1.67^bc^	2
ÇNK-6	Moderate foliar, wrinkling	1.67^bc^	2
ÇNK-8	Mild foliar	1.00^cd^	1
YLV-14	Non-symptomatic	0.33^d^	0
YLV-28	Moderate foliar, wrinkling	1.67^bc^	2
YLV-31	Mild foliar	1.00^cd^	1
YLV-32	Mild foliar	1.33^bcd^	1
Kantar-05*	Severe disortion and malformation	3.00^a^	3
Yakutiye-98*	Non-symptomatic	0.33^d^	0

*F value = 7.35, (*p* < 0.01), LSD_0,01_ = 1.03, *:control cultivars.

### 3.2 SYBR green qRT-PCR and primer efficiency

**FIGURE 3:**
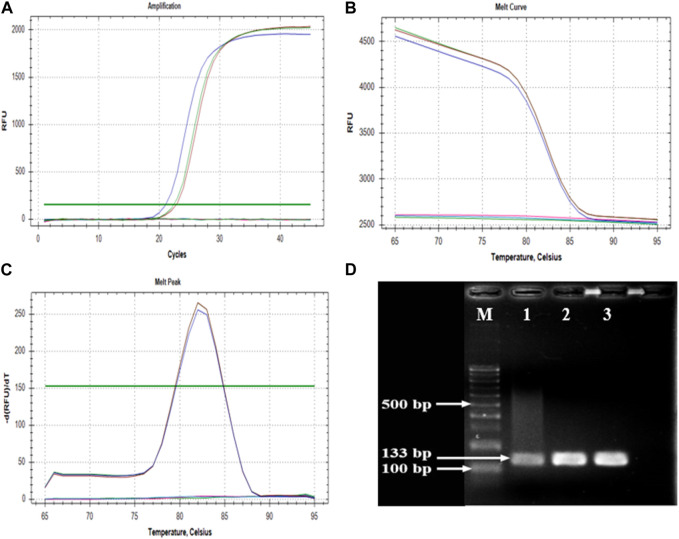
Parameters of amplification and melting analyses. **(A)**; sharp amplification peak confirms the positivity, **(B,C)**; melting curve results indicates the specificity, **(D)**; agarose gel photo of an amplified 133 bp product. M: GeneRuler 100 bp DNA Ladder (Thermo Fischer Scientific, Waltham, MA, United States), 1, 2, and 3: cDNAs of BRS-22 genotype in three replicate.

**FIGURE 4:**
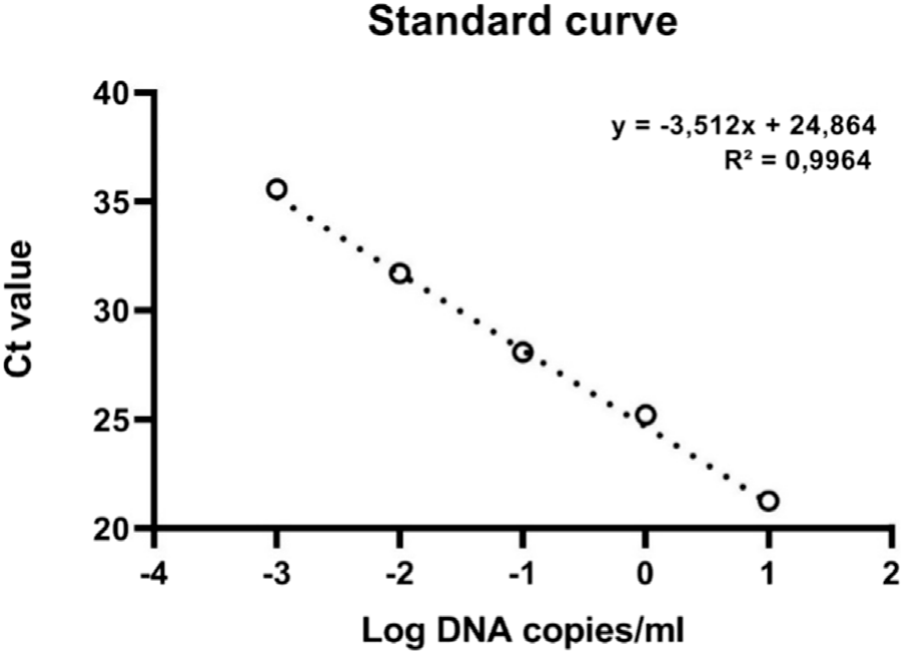
The standard curve of qRT-PCR of BCMV.

The primer set CPF02F (5′-ATC​GGA​TCG​AGC​AAG​AGA​AGC-3′) and CPF02R (5′-GTC​CCT​TGC​AGT​GTG​CCT​TT-3′) showed a single amplicon (Figure 3A) with a sharp melt curve and were selected for thorough testing in the present study. The primers amplified a 133 bp product from the tissues from BCMV inoculated plants but not from uninoculated controls plants. No primer dimers were detected in the melting curve analysis (Figure 3B). Melting curve analysis showed that the tested primers amplified a single peak with a distinct melting point of 82°C in case of the presence of BCMV RNA (Figure 3C). The amplified products were confirmed by agarose gel electrophoresis (Figure 3D). The efficiency of the primer set was calculated from the standard curve slopes using the CFX Maestro Software and visualized using GraphPad Prism. The cDNA dilution series indicated high efficiency (0.93) and high linearity with R2 > 0.99 (Figure 4).

### 3.3 Determination of viral load by qRT-PCR

**FIGURE 5:**
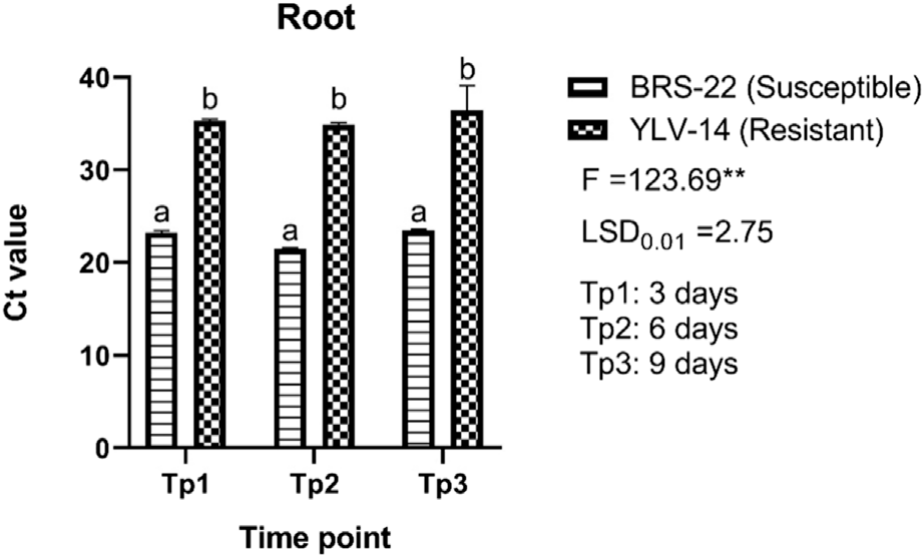
The levels of accumulation of BCMV by qRT-PCR in the roots of BRS-22 and YLV-14 genotypes at different time points. Levels not connected by the same letter are significantly different Ct values between genotypes based on LSD test at the 1% level.

Trifoliate leaves and roots of BCMV inoculated plants were sampled to find out the viral accumulation at different time points from BRS-22 and YLV-14 genotypes. The significant differences among Ct values were observed between genotypes at each time point regardless of tissue. The variation in Ct values of root tissue of susceptible and resistant genotypes was significant according to the ANOVA results (F = 123.69; LSD_0.01_ = 2.75) (Figure 5). The level of Ct values of the amplicon determining BCMV at 3-, 6-, and 9-days pi was found as 23.21, 21.47, and 23.47 for root tissues of BRS-22, respectively. Besides, the level of Ct values was determined as 35.28 at 3 days, 34.88 at 6 days, and 36.44 at 9 days for root tissues of YLV-14. However, there was no significant variation among Ct values at different time points for each genotype. The Ct values of BCMV-infected susceptible and resistant genotype’s roots were separated and placed in the same groups according to the LSD_0.01_ test. Generally, the Ct values of the susceptible genotype at different time points were lower than the resistant genotype’s Ct values. These results revealed that the accumulation of BCMV NL-4 strain in the root tissues was considerably higher in BRS-22 than in YLV-14.

**FIGURE 6:**
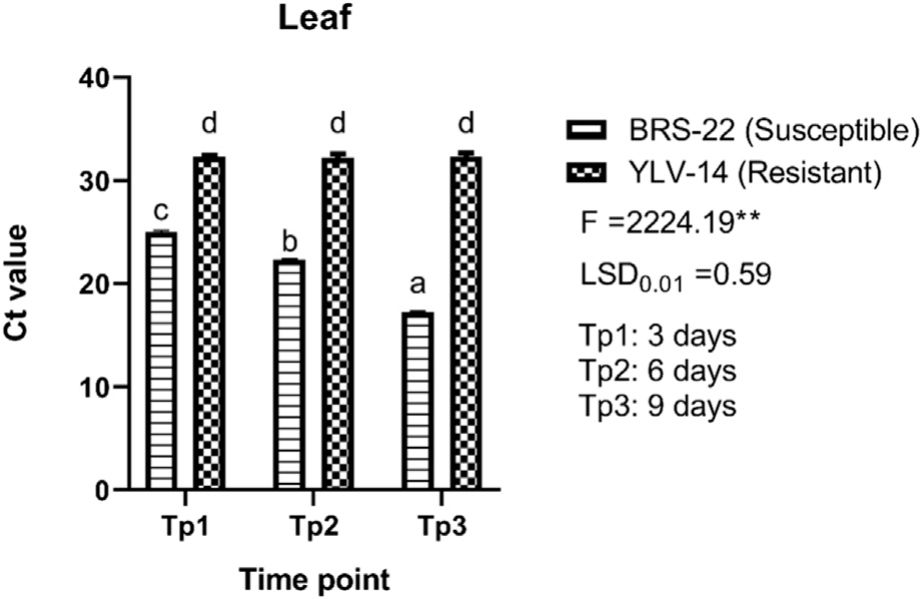
The levels of accumulation of BCMV by qRT-PCR in the leaves of BRS-22 and YLV-14 genotypes at different time points. Levels not connected by the same letter are significantly different Ct values between genotypes based on LSD test at the 1% level.

For leaf tissues of genotypes, there were also found significant differences among Ct values, (F = 2224.19; LSD_0.01_ = 0.59) (Figure 6). The Ct values ranged from 17.19 (BRS-22, 9 days pi) to 32.32 (YLV-14, 9 days pi) for leaf tissues. The level of Ct values for leaf tissues of BRS-22 gradually and significantly decreased at each time point during BCMV infection, which indicates a sharp increase in virus accumulation. No significant variations among Ct values for leaf tissues were recorded at different time points for YLV-14 after inoculation. All Ct values obtained from leaf tissues of YLV-14 at different time points were placed statistically in the same group. Here, these variations between the Ct values of susceptible and resistant genotypes for BCMV accumulation were consistent with the result of the pathogenicity test.

## 4 Discussion

Sustainable agriculture and crops are affected by abiotic and biotic stresses, including viruses, that cause millions of dollars’ worth of yield losses every year (Mumford et al., 2016; Yılmaz et al., 2021). Identifying plants infected with new viruses and the increase of virus population in agricultural areas or a host are major factors in controlling plant viruses. Disease management strategies such as cultivating resistance varieties, controlling plant material transfer, and pursuing new variants are generally preferable strategies to reduce the harms caused by plant-pathogenic viruses (Anderson et al., 2004; Jones, 2009; Elena et al., 2014).

BCMV is one of the most prevalent viruses infecting common beans and a variety of other legume hosts (Morales, 2006). The yield reduction caused by BCMV might reach up to 100% (Damayanti et al., 2008; Saqib et al., 2010; Singh and Schwartz, 2010; Verma and Gupta, 2010; Li et al., 2014). The use of resistant cultivars to the pathogen plays an active role in reducing the virus’s damages. In the present study, twenty common bean genotypes and two check lines were inoculated with the BCMV NL-4 strain to understand the host suitability against the pathogen. Inoculated common bean genotypes exhibited various symptoms, including wrinkling, mosaic, and dwarfing with severe distortion and malformation changing on a scale from mild foliar to intense mosaic. Several types of symptoms in literature have been reported on BCMV infected common beans, such as mosaic, leaf malformation, stunting (Drijhout et al., 1978), vein clearing, mottling, leaf curling, and chlorosis (Morales and Bos, 1988; Kılıç et al., 2020). The symptoms observed in this study on BCMV-infected genotypes were in accordance with the symptom types previously reported in the studies mentioned above. In addition, this paper has figured out the current position of 20 advanced common bean genotypes demonstrating resistance and susceptibility to BCMV, which were conducted in line with previous studies to assess the presence of BCMV resistance in different genotypes (Mukeshimana et al., 2005; Bello et al., 2014; Boersma et al., 2014; Yeken et al., 2018; Palacıoğlu et al., 2021). The promising genotype YLV-14 resistant to BCMV NL-4 strain can also be evaluated in various classical and molecular breeding studies such as crossing, QTLs, MAS, GWAS, and functional genomics to accelerate the crop improvement. To the best of our knowledge, there was no developed scale related to BCMV symptom severity on host plants. Here, we adapted for the first time a 0–3 scale developed for Yam mosaic virus (YMV) by Odu et al. (2004) to successfully classify the symptom severity of BCMV. Mangeni et al. (2020) also used this scale in determining the disease severity of BCMNV, another *Potyvirus* disease of common bean, and these results are consistent with this study reported here.

Plant RNA viruses have the indisputable potential for genetic variety, fast development, and adaptability to new environmental conditions (Abadkhah et al., 2020). BCMV, a typical member of *Potyviruses*, has all the features of the genus, such as high mutation ability, recombination, and formation of various strains (Gibbs et al., 2020). Previous studies identified different pathogroups and strains in BCMV classification (Feng et al., 2015). When the nucleotide sequences of all strains and pathogroups of BCMV in the GenBank database were downloaded and aligned, it was considered that it would be difficult to design an SYBR Green-based real-time PCR primer sets that would enable the amplification of all strains due to the limited conserved genomic regions. Therefore, we used a BCMV NL-4 isolate, whose CP sequence information is available in GenBank (OL741709), to stimulate bean-BCMV interaction and investigate viral accumulation in root and leaf tissues of susceptible and resistant genotypes.

Identifying viruses at the molecular level is unquestionably important in plant virology. A huge number of papers have focused on the design, development, and validation of PCR-derived techniques, with a particular emphasis on real-time PCR in recent years (Olveria et al., 2021). Several studies have been reported to detect BCMV using different techniques such as DAS-ELISA, RT-PCR, and sequencing (Vetten et al., 1992; Xu and Hampton, 1996; Arlı-Sökmen et al., 2016). However, no publications are available for developing a qRT-PCR assay to detect and quantify BCMV in common bean. Here, we established an SYBR Green-based qRT-PCR methodology for BCMV-NL-4 detection and quantification for the first time. This novel developed an assay for BCMV detection has simplified viral quantification, which is increasingly gaining importance as a basic supplement in research, especially early detection of the pathogen. The developed qRT-PCR assay provided various advantages over traditional RT-PCR assays, including increased sensitivity, specificity, and quantitative quantification, as well as ease of standardization for the evaluation of these genotypes. The results that related to the specificity, sensitivity, and quantification in BCMV detection were in accordance with the previous qRT-PCR assays developed for other plant viruses (Mumford et al., 2000; Picó et al., 2005; López et al., 2006; Ling et al., 2007; Hongyun et al., 2008; Ruiz-Ruiz et al., 2009a; Debreczeni et al., 2011; Ferriol et al., 2011; Sharma and Dasgupta, 2012; Herrera-Vásquez et al., 2015; MacKenzie et al., 2015). The designing of primers is a key factor for a sequence-based detection system, and the CP gene is a good target for viral detection due to its specificity (Ghosh et al., 2020). The qRT-PCR assay established in this study amplified a 133 bp fragment of CP of BCMV-NL4 that was used to quantify BCMV in leaves and roots at different time points. We use SYBR Green technology based on binding the fluorescent dye to dsDNA since it is easy to use and relatively cost-benefit than TaqMan technology based on exonuclease activity of Taq polymerase enzyme and dual-labelled oligonucleotide (Mahmoudian et al., 2011). The primers utilized in the assay effectively amplified the target gene, and the results showed that SYBR Green-based qRT-PCR is a fast and sensitive way of directly detecting and quantifying BCMV from leaves and roots. The newly designed primer set targeting the CP of BCMV NL-4 allowed the detection of BCMV with satisfactory E, R2, and slope values, which are reliable with the established standards (Broeders et al., 2014), verifying the accuracy and linear response of the experiments over a wide range of dilutions and implying the absence of PCR inhibitors.

It is often time-consuming and difficult to discover resistance genes, establishing long-period pathogenicity tests, especially when the BCMV mosaic strains employed as a screening inoculum belong to pathogenicity groups other than those identified by Drijfhout (1978) and Feng et al. (2018). In the current study, it is considered that the accumulation of the pathogen in the initial stage of infection can provide valuable information for the selection of cultivars. Based on this data, we imagined that the severity of symptoms exhibited by infected plants might be related to changes in genomic regions of pathogens associated with replication, virus movement, and processes that control the level of virus accumulation in infected plants. Significant differences were found between Ct values obtained at different time points in root and leaf tissue of susceptible and resistant genotypes. Mainly, the Ct values were found to be higher in the resistant genotype than the susceptible genotype in both tissues. The presence of the pathogen in the root 3 days pi in both susceptible and resistant cultivars undoubtedly indicates that the pathogen causes systemic infection. Agrios (2005) stated that when a virus infects a plant, it takes 2–5 days or more for the virus to travel out of an infected leaf, and once in the phloem, the virus spreads quickly toward developing areas (apical meristems) or other food-utilizing sections of the plant, such as tubers and rhizomes. The Ct values obtained from both roots and leaves confirm active viral movement in the plant in accordance with Agrios (2005), showing the pathogen is moving in the phloem. Some papers have been reported indicating the viral movement is triggered by viral determinants of phloem viruses. Following the symptom onset and viral accumulation, Roberts et al. (2007) proved a close link between cauliflower mosaic virus (CaMV) infection and patterns of photoassimilate distribution in sink organs, indicating the virus movement. Similarly, melon necrotic spot virus (MNSV) first moves from the cotyledons to the roots *via* the external phloem before moving to the shoot apex *via* the internal phloem (Gosalvez-Bernal et al., 2008). In contrast, some host determinants promote or restrict virus movement in the phloem. Host factors, in addition to viral components, can be recruited to aid virus phloem movement. Cellular proteins may be involved in the creation of viral complexes and can promote the effective transport of such complexes, as well as acting as stabilizing proteins or as protective agents against plant defence systems (Hipper et al., 2013).

The qRT-PCR results of this study revealed that BRS-22 had more accumulation of BCMV NL-4 strain than YLV-14 at different time points in both tissues. This scenario is thought to be generated by the plant’s natural defensive system or by the features of the variety, as stated in Hipper et al. (2013). For CVYV in cucumber, real-time PCR yielded repeatable findings of susceptible and resistant landraces, and the quantity of target viral load detected in genotypes was significantly varied, demonstrating variances in viral accumulation linked to their different levels of resistance (Picó et al., 2005). Jiang et al. (2013) measured the viral load of HTNV (Hantaan virus) by using an SYBR Green I-based one-step qRT-PCR technique and observed low viral titres in samples with high Ct values. Campos et al. (2019) reported that qRT-PCR based on SYBR Green methodology produced high Ct values in low titres of olive viruses. Similarly, de Freitas-Vanzo et al. (2021) used the qRT-PCR to quantify BGMV titre in host plants and found variations in symptom expression and BGMV titre between cultivars and lineages at 10 and 25 pi and the bean cultivar “Tangará” as reported in YLV-14 in this research, had a higher Ct value but showed less symptom severity than the other cultivars. Similar studies have also been carried out to investigate viral load that indicated the resistance and susceptibility of many hosts against various plant viruses, including BYDV, WYMV, TSWV, CBSD, and various grapevine viruses, which results were in agreement with the outcomes reported in this study (Gil salas et al., 2009; Jarošová and Kundu, 2010; Liu et al., 2013; Soler et al., 2015; Fajardo et al., 2017; Shirima et al., 2017). Based on this data, the established assay provides a reliable quantitative tool for accumulating BCMV in the tissues of susceptible and resistant genotypes at various time periods.

In summary, the developed qRT-PCR provided a high degree of reliability, sensitivity, and reproducibility for the prediction of virus accumulation in infected susceptible and resistant cultivars as an effective tool for early detection of diseases, enabling to predict of the genotypes in the early stage of infection. This novel assay may detect viral load differences between highly susceptible and non-symptomatic genotypes and may have utility for plant breeding systems. The results of the study will provide new insights into host-pathogen interactions. In addition, this method will contribute significantly to detection methods for BCMV in different research areas and will provide a reference for future studies.

## Data Availability

The original contributions presented in the study are included in the article/Supplementary Material, further inquiries can be directed to the corresponding authors.
