# Supplementary material for: A novel study on bean common mosaic virus accumulation shows disease resistance at the initial stage of infection in Phaseolus vulgaris
Source: Front Genet. 2023 Mar 20;14:1136794. doi: 10.3389/fgene.2023.1136794 (PMC10067576; doi:10.3389/fgene.2023.1136794)
Supplement: Supplementary file 1 [file Table1.docx]

Supplementary Material

A novel research on bean common mosaic virus accumulation shows disease resistance at the initial stage of infection in *Phaseolus vulgaris*

Ali ÇELİK^1*^, Orkun EMİRALİOĞLU^2^, Mehmet Zahit YEKEN^2^, Vahdettin ÇİFTÇİ^2^, Göksel ÖZER^1*^ Yoonha Kim^3^, Faheem Shehzad BALOCH^*4^ and Yong Suk Chung^*5^

*** Correspondence:** Ali Çelik
[alicelik032@gmail.com](mailto:alicelik032@gmail.com)

Göksel Özer

[gokozer@gmail.com](mailto:gokozer@gmail.com)

Faheem Shehzad Baloch

[balochfaheem13@gmail.com](mailto:balochfaheem13@gmail.com)

Yong Suk Chung

[yschung@jejunu.ac.kr](mailto:yschung@jejunu.ac.kr)

# Supplementary Data

**SI 1.** The advanced genotypes used in this study that previously characterized in terms of morphologically and agronomically according to IPGRI (International Plant Genetic Resources Institute) and EU-CPVO (European Union Community Plant Variety Office).

| **Number** | **Lines** | **Province** | **Town** | **Village** | **Altitude** | **Coordinates** |
| --- | --- | --- | --- | --- | --- | --- |
| 1 | YLV-14 | Yalova | Çiftlikköy | Kabaklı | 125 | 40°39´33.4188"N/29°24´36.1908"E |
| 2 | YLV-28 | Yalova | Merkez | Kurtköy | 362 | 40°33'12.70"N/29°12'52.17"E |
| 3 | YLV-31 | Yalova | Merkez | Hacımehmet | 70 | 40°36'56.2320''N/29°14'37.6224''E |
| 4 | YLV-32 | Yalova | Merkez | Sugören | 428 | 40°33'38.32"N/29°19'34.07"E |
| 5 | BLKSR-3 | Balıkesir | Manyas | Salur Mh. | 29 | 40° 5'58.61"N/27°56'16.65"E |
| 6 | BLKSR-4 | Balıkesir | Manyas | Akçaova Mh. | 30 | 40° 7'16.68"N/27°51'15.26"E |
| 7 | BLKSR-19 | Balıkesir | Sındırgı | Kürendere | 1051 | 39°18'52,7"N/28°32'53,1"E |
| 8 | BRS-3 | Bursa | Yenişehir | Osmaniye | 377 | 40°10'18.45"N/29°37'15.12"E |
| 9 | BRS-4 | Bursa | Inegöl | Cerrah | 327 | 40°4'18.8364''N/29°26'47.7600''E |
| 10 | BRS-21 | Bursa | Kestel | Kızılören | 435 | 40° 7'39.19"N/ 29°21'9.43"E |
| 11 | BRS-22 | Bursa | Kestel | Aksu | 360 | 40°10'2.02"N/ 29°18'58.01"E |
| 12 | BRS-23 | Bursa | Kestel | Aksu | 360 | 40°10'2.02"N/ 29°18'58.01"E |
| 13 | BRS-24 | Bursa | Orhaneli | Küçükorhan | 487 | 39° 48' 8.9640''N/29° 2' 7.4544''E |
| 14 | DZC-2 | Düzce | Merkez | Derdin | 859 | 40°42´28.4292"N/31°13´33.1608"E |
| 15 | DZC-3 | Düzce | Merkez | Derdin | 859 | 40°42´28.4292"N/31°13´33.1608"E |
| 16 | BLCK-7 | Bilecik | Pazaryeri | Dereköy | 876 | 39°59'12.52"N/ 29°51'7.17"E |
| 17 | ÇNK-2 | Çanakkale | Yenice | Çınarcık | 320 | 39°57'6.22"N/ 27°10'54.75"E |
| 18 | ÇNK-4 | Çanakkale | Biga | Aşağıdemirci | 25 | 40°14'38.70"N/ 27°22'17.65"E |
| 19 | ÇNK-6 | Çanakkale | Biga | Gerlengeç | 25 | 40°17'26.36"N/ 27°25'14.56"E |
| 20 | ÇNK-8 | Çanakkale | Bayramiç | Beşik | 100 | 39°44'15.48"N/ 26°41'34.82"E |

**SI 2**. Primers designed targeting the CP gene (accession: OL741709) of BCMV for real-time PCR assay.

| **Primer pair 1** | | | | | |
| --- | --- | --- | --- | --- | --- |
|  | **Sequence (5'->3')** | **Length** | | **Tm** | **GC%** |
| **F** | TCGGATCGAGCAAGAGAAGC | 20 | | 59.90 | 55.00 |
| **R** | ACCCAATCTTTACTGCGGGG | 20 | | 60.03 | 55.00 |
| **bp** | 189 | | | | |
| **Primer pair 2** | | | | | |
|  | **Sequence (5'->3')** | **Length** | | **Tm** | **GC%** |
| **F** | TTCCGAGAGACCGTACATGC | 20 | | 59.55 | 55.00 |
| **R** | TGTTAACGTTGCTGAGGGCT | 20 | | 59.89 | 50.00 |
| **bp** | 164 | | | | |
| **Primer pair 3** | | | | | |
|  | **Sequence (5'->3')** | **Length** | | **Tm** | **GC%** |
| **F** | GGTAACGTGGCAACAACCAG | 20 | | 59.69 | 55.00 |
| **R** | CCCAATCTTTACTGCGGGGA | 20 | | 59.75 | 55.00 |
| **bp** | 107 | | | | |
| **Primer pair 4** | | | | | |
|  | **Sequence (5'->3')** | **Length** | | **Tm** | **GC%** |
| **F** | CTGAAAGGCACACTGCAAGG | 20 | | 59.69 | 55.00 |
| **R** | CGACGCGAGATGCTAACTGT | 20 | | 60.52 | 55.00 |
| **bp** | 109 | | | | |
| **Primer pair 5** | | | | | |
|  | **Sequence (5'->3')** | **Length** | | **Tm** | **GC%** |
| **F** | GTAACGTGGCAACAACCAGC | 20 | | 60.32 | 55.00 |
| **R** | ACGCGAGATGCTAACTGTGA | 20 | | 59.47 | 50.00 |
| **bp** | 134 | | | | |
| **Primer pair 6** | | | | | |
|  | **Sequence (5'->3')** | **Length** | | **Tm** | **GC%** |
| **F** | TGGCAACAACCAGCGAGAAT | 20 | | 60.54 | 50.00 |
| **R** | AGCCCATGCCAAGAAGTGTA | 20 | | 59.30 | 50.00 |
| **bp** | 81 | | | | |
| **Primer pair 7** | | | | | |
|  | **Sequence (5'->3')** | **Length** | | **Tm** | **GC%** |
| **F** | CCGAGAGACCGTACATGCC | 19 | | 59.93 | 63.16 |
| **R** | TGCTTCTCTTGCTCGATCCG | 20 | | 60.18 | 55.00 |
| **bp** | 122 | | | | |
| **Primer pair 8** | | | | | |
|  | **Sequence (5'->3')** | **Length** | | **Tm** | **GC%** |
| **F** | GCAGTAGCACAGATGAAGGC | 20 | | 58.99 | 55.00 |
| **R** | ATTCTCGCTGGTTGTTGCCA | 20 | | 60.54 | 50.00 |
| **bp** | 90 | | | | |
| **Primer pair 9** | | | | | |
|  | **Sequence (5'->3')** | **Length** | | **Tm** | **GC%** |
| **F** | TCCAAAACATCGGATCGAGCA | 21 | | 60.07 | 47.62 |
| **R** | CTGTTAACGTTGCTGAGGGC | 20 | | 59.48 | 55.00 |
| **bp** | 71 | | | | |
| **Primer pair 10** | | | | | |
|  | **Sequence (5'->3')** | **Length** | | **Tm** | **GC%** |
| **F** | GCACACTGCAAGGGACGTTA | 20 | | 60.88 | 55.00 |
| **R** | CAATCTTTACTGCGGGGAGC | 20 | | 58.98 | 55.00 |
| **bp** | 81 | | | | |
| **Primer pair 11** | | | | | |
|  | **Sequence (5'->3')** | **Length** | **Tm** | | **GC%** |
| **F** | ATCGGATCGAGCAAGAGAAGC | 21 | 60.27 | | 52.38 |
| **R** | GTCCCTTGCAGTGTGCCTTT | 20 | 61.11 | | 55 |
| **bp** | 133 | | | | |
